# Are experienced and high-level race walking athletes able to match pre-programmed with executed pacing?

**DOI:** 10.1590/1414-431X20198593

**Published:** 2019-06-03

**Authors:** D.L. Alves, R. Cruz, A.E. Lima-Silva, P.R. Domingos, R. Bertuzzi, R. Osiecki, F.R. De-Oliveira, J.R.P. Lima

**Affiliations:** 1 Centro de Estudos da Performance Física (CEPEFIS), Escola de Educação Física e Esporte, Universidade Federal do Paraná, Curitiba, PR, Brasil; 2 Grupo de Estudos em Desempenho Aeróbio (GEDAE), Departamento de Esporte, Escola de Educação Física e Esporte, Universidade de São Paulo, São Paulo, SP, Brasil; 3 Grupo de Pesquisa em Performance Humana (GPPH), Universidade Tecnológica Federal do Paraná, Curitiba, PR, Brasil; 4 Faculdade de Educação Física e Esporte, Universidade Federal de Juiz de Fora, Juiz de Fora, MG, Brasil; 5 Departamento de Educação Física, Universidade Federal de Lavras, Lavras, MG, Brasil

**Keywords:** Aerobic evaluation, Performance, Sport, Elite athlete, Physiology

## Abstract

The objective of this study was to verify the agreement between pre-programmed and executed pacing during race walking and whether level of the athletes experience and performance influenced this relationship. Twenty-nine national and international race walkers participated in this study (14 males, 24.0±7.1 years old, and 15 females, 23.3±7.3 years old). Pre-programmed pacing for 10- and 20-km official walking races was self-selected via demonstrative pacing charts prior to races, while executed pacing was analyzed by a specialist investigator via an individual plot of current velocity versus distance. There was no agreement between pre-programmed and executed pacing (P=0.674). There was no association between the ability to match the pre-programmed pace with the executed pace and race walking experience or level of performance. Low- and high-performance athletes pre-programmed a similar pacing profile (P=0.635); however, high-performance athletes generally executed an even pacing strategy, while low-performance athletes generally adopted a positive pacing strategy (P=0.013). Race walkers did not faithfully match their pre-programmed with their executed pacing, and this seemed to be independent of previous experience and level of performance. High-performance athletes, however, tended to execute an even pacing strategy, even though this had not been pre-programmed.

## Introduction

Pacing is defined as the alterations in power output or velocity that occur throughout a race, in order to reach the endpoint in the shortest possible time (1–4). This is an important determinant of performance in endurance sports such as cycling, speed skating, kayaking, running, and race walking (5–9). In particular, for race walking ranging from 10 to 20 km, a negative pacing (i.e., a slower start followed by a progressive increase in velocity as the race progresses) seems to be the most used pacing strategy (10–12). However, athletes may individually choose other pacing profiles, such as even (maintaining a constant velocity), positive (gradually decreasing the velocity over the distance), parabolic (starting with high velocities, slowing down during mid-race and increasing at the end), and variable (there is no clearly defined pattern for velocity distribution) (1,2,13,14). However, it is currently unknown whether the athletes follow a pre-programmed pacing profile or they “unconsciously” execute a given pacing during race walking.

Pacing may be influenced by learning and experience (3,15–17). For example, Hopkins and Hewson (18) reported that variations in end times for different running distances (3000 m to marathon) tend to be smaller for experienced than for inexperienced athletes. Foster et al. (19) also verified the effect of learning on performance during 3-km cycling and 2-km rowing time trials and reported a progressive increase in power output during the initial and middle phases of the race as individuals were repeating the trials. Together, these studies suggest that previous experience with endurance events is essential for determining optimal pacing and ultimately maximizing overall performance. Experience may be more important in sports demanding more technical skills as race walking; more experienced athletes may have a lower deterioration in walking technique and consequently a lower number of warnings (11,12,20–22). Thus, experience in race walking might have an impact on the ability of the athlete to execute a given pre-programmed pacing profile.

Another important factor that might impact pacing is the level of performance. It has been demonstrated that medalists in the 20- and 50-km races of the 7th World Race Walking Cups started the race at velocities lower than their best personal mark, while non-medalists started the race at velocities higher than their best personal mark (12). However, it is not known whether level of performance influences pacing by allowing athletes to execute precisely a pre-programmed pacing profile or by enabling them to react more efficiently to unpredictable challenges demanding a momentary pacing alteration during races (e.g., opponent scape).

Understanding whether an actual pacing profile differs from the pre-programmed profile by the athlete and whether different levels of experience and performance influence the ability to execute a pre-established pacing strategy can provide insights into how pacing is regulated during race walking. Therefore, the objective of this study was to verify the agreement between pre-programmed and executed pacing during official 10- and 20-km race walking, as well as to verify whether prior experience and level of performance of the athletes influenced this relationship. We hypothesized that more experienced and high-performance athletes better matched a pre-programmed pacing with their executed pacing compared with the less experienced and low-performance racing competitors.

## Material and Methods

### Participants

Twenty-nine race walkers of national and international experience levels participated in this study (14 males and 15 females, 24.0±7.1 and 23.3±7.3 years old, respectively). Athletes were in the under 20 (4 males and 7 females) and adult (10 males and 8 females) categories, and participated in 10- and 20-km races, respectively. Athletes at the national level had participated in state and national competitions, while athletes at the international level had participated in competitions such as the Olympic Games, South American/Pan American Championships, IAAF World Race Walking Cup, and competitions hosted in other countries in the last four years before the study. One athlete ranked third in the 2017 IAAF World Championships and was among the top 10 race walking athletes in the world. All participants received a verbal explanation about the potential benefits, risks, and discomfort associated with this study. Each was asked to give written informed consent before participating in the study. This study was approved by the Ethics Committee of Universidade Federal de Juiz de Fora (1.047.279) and was performed in accordance with the ethical standards established by the Declaration of Helsinki (23).

### Experimental design

The data were collected during the 10- and 20-km races of the largest Brazilian race walking competition, which was organized by the Brazilian Athletics Confederation (CBAt) and followed the rules established by the International Association of Athletics Federations (IAAF). The races were performed on a 1-km flat street circuit (altitude 17.0±3.0 m). The time recorded for each lap of 1 km was provided by the official organizer (CBAt).

### Instruments and procedures

**Figure 1:**
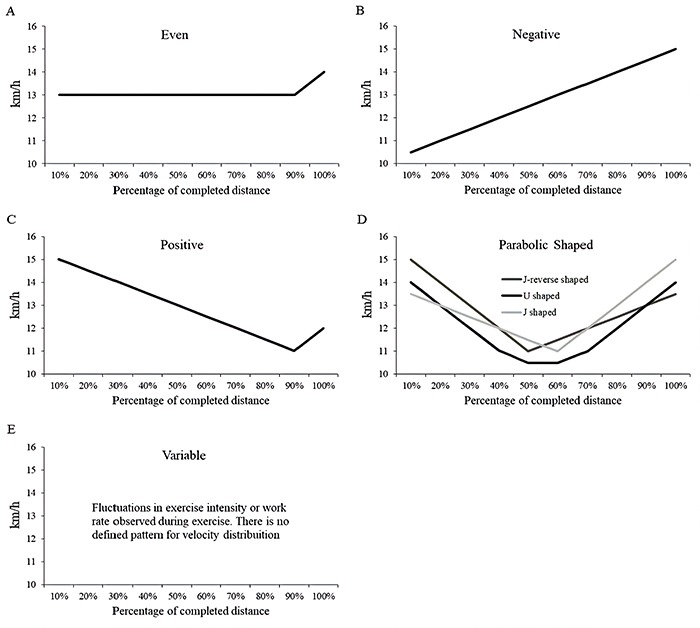
Demonstrative chart that athletes used to pre-program the pacing for the race.

A dashboard with the most common pacing profiles during race walking (i.e., even, positive, negative, parabolic, or variable) was prepared and showed to each athlete before the competition. The dashboard was created based on a previous study that described various types of pacing strategy in athletic competitions (1). The pacing profiles were displayed as velocity (km/h) for each 10% of the total distance. Athletes were familiarized prior to responding to the questionnaire through examples of pacing strategy performed in previous competitions of endurance sports. In addition, verbal explanations were provided on how to interpret figures and then the participants indicated which strategy they would use in that competition. The pacing profiles were explained as: a) even pacing, where the athlete maintains (or changes minimally) the velocity during the race; b) positive pacing, where the athlete starts at a high velocity and decreases gradually during the race; c) negative pacing, where the athlete starts at low velocity and gradually increases during the race; d) parabolic pacing, where the athlete starts at high velocity, decreases during the race, and increases at the end; and e) variable pacing, where there is no defined pattern for velocity distribution (Figure 1).

After athletes had indicated their pre-programmed pacing strategy, they warmed up for approximately 40 min before speed walking their respective races. For the executed pacing, the velocity for each lap (km/h) was plotted against each 10% of the total distance and displayed in graphs. A specialist investigator, who was an expert in race walking and not aware of the pre-programmed pacing strategy of the athletes, classified each executed pacing profile as one of the five possible patterns.

The experience was measured by recording how long (in months) he/she had been practicing race walking up to the day of competition. Then, the sample was split into two groups using the 50th percentile, one group with less experience (5–48 months) and the other group with more experience (49–240 months). The level of performance was evaluated similarly by dividing the athletes into two groups using the 50th percentile, one with high (1st to 5th place) and another with low (>6th place) level of performance in the race.

### Statistical analysis

Data are reported as means±SD and frequency. The data normality assumption was evaluated by the Shapiro-Wilk test. The level of agreement between pre-programmed and executed pacing was verified using the Kappa agreement test. In order to test the relationship between experience/level of performance with the agreement of pre-programmed and executed pacing, a nominal variable was created to classify athletes as performing or not the pre-programmed pacing during the event. For athletes who did not match the pre-programmed pacing with their executed pacing, code 1 was assigned. Those for whom agreement was obtained, code 2 was assigned. The association of experience and level of performance with agreement in pacing strategy was tested with Fisher’s exact test. The association of experience and level of performance with pre-programmed and executed pacing was tested with the chi-squared test. All analyses were performed using the Statistical Package for the Social Sciences (SPSS) version 20.0 (IBM Corp., USA). Statistical significance was considered when P<0.05.

## Results

The average velocity was 10.59±0.74 km/h in 10-km and 11.09±1.59 km/h in 20-km races. The frequency of pre-programmed and executed pacing strategies is shown in Table 1. The most frequent pre-programmed pacing strategy was the negative (n=16, 55.2%), followed by the even (n=10, 34.4%), positive (n=2, 6.8%), and parabolic (n=1, 3.4%). The most frequently executed pacing strategy was the positive (n=14, 48.2%), followed by the even (n=12, 41.3%), parabolic (n=2, 6.9%), and negative (n=1, 3.4%). None of the participants pre-programmed or executed the variable pacing strategy. The agreement analysis indicated that there was no significant association between pre-programmed and executed pacing (k=–0.074; P=0.307; n=29).

**Table 1:** Number and percentage of pre-programmed *vs* executed pacing.

Pre-programmed pacing	Executed pacing			
	Even	Positive	Negative	Parabolic
Even (n=10)	3 (10.3%)	6 (20.7%)	1 (3.4%)	0 (0%)
Positive (n=2)	1 (3.4%)	1 (3.4%)	0 (0%)	0 (0%)
Negative (n=16)	8 (27.6%)	6 (20.7%)	0 (0%)	2 (6.9%)
Parabolic (n=1)	0 (0%)	1 (3.4%)	0 (0%)	0 (0%)

Statistical test: Kappa (k=–0.074; P=0.307; n=29).

**Figure 2:**
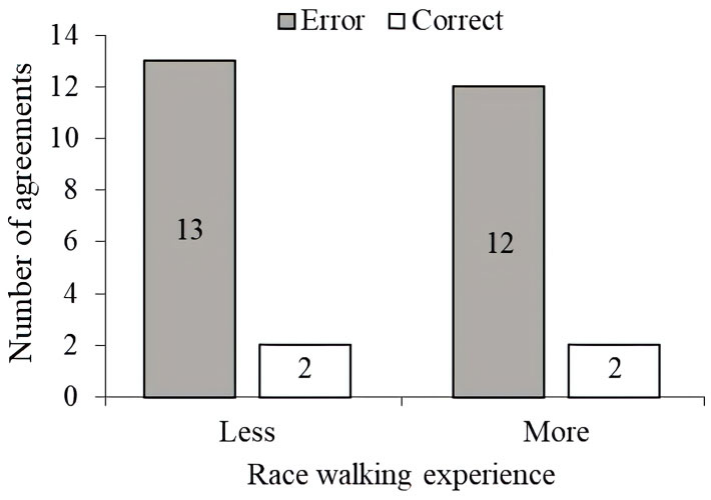
Agreement between pre-programmed and executed pacing in less and more experienced race walkers (Fisher’s exact test, P=0.99).

As shown in Figure 2, level of experience was not significantly related to the agreement between pre-programmed and executed pacing (P=0.99). In addition, more experienced athletes did not have a clear preferable pre-programmed or executed strategy for pacing nor did they differ from less experienced athletes (pre-programmed *χ* ^2^(3)=3.370, P=0.338; executed *χ* ^2^(3)=4.447, P=0.217, Table 2).

**Table 2:** Pre-programmed and executed pacing in less and more experienced race walkers.

	Pre-programmed pacing		Executed pacing	
	Less	More	Less	More
Even	4 (26.7%)	6 (42.9%)	5 (33.3%)	7 (50.0%)
Positive	2 (13.3%)	0 (0%)	9 (60.0%)	5 (35.7%)
Negative	8 (53.3%)	8 (57.1%)	1 (6.7%)	0 (0%)
Parabolic	1 (6.7%)	0 (0%)	0 (0%)	2 (14.3%)

Statistical test: chi-squared (pre-programmed *χ* ^2^(3)=3.370, P=0.338; executed *χ* ^2^(3)=4.447, P=0.217).

**Figure 3:**
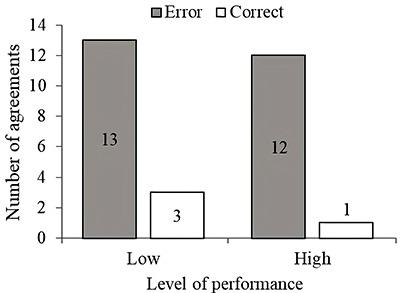
Agreement between pre-programmed and executed pacing in athletes with low and high levels of performance (Fisher’s exact test, P=0.606).

As shown in Figure 3, the level of performance was also not related to the match between pre-programmed and executed pacing (P=0.606). In addition, high-performance athletes did not have a clear preferable pre-programmed pacing strategy, and thus did not differ from low-performance athletes (pre-programmed *χ* ^2^(3)=1.708, P=0.635). However, high-performance athletes predominantly used even pacing, while low-performance athletes predominantly used positive pacing (executed *χ* ^2^(3)=10.709, P=0.013, Table 3).

**Table 3:** Pre-programmed and executed pacing in low- and high-performance race walkers.

	Pre-programmed pacing		Executed pacing	
	Low	High	Low	High
Even	5 (38.5%)	5 (31.2%)	2 (15.4%)	10 (62.5%)*
Positive	1 (7.7%)	1 (6.3%)	10 (76.9%)	4 (25.0%)*
Negative	6 (46.1%)	10 (62.5%)	1 (7.7%)	0 (0%)
Parabolic	1 (7.7%)	0 (0%)	0 (0%)	2 (12.5%)

Statistical test: chi-squared (pre-programmed *χ* ^2^(3)=1.708, P=0.635; executed *χ* ^2^(3)=10.709, P=0.013). *Significantly different from low-performance athletes for executed pacing (P=0.013).

## Discussion

This is the first study investigating the agreement between pre-programmed and executed pacing in race walking, and whether experience and level of performance influenced this relationship. Our findings indicated that the majority of athletes pre-programmed the negative (55.2%) or even (34.4%) pacing strategy; however, they were unable to follow this pre-programmed pacing strategy and executed either the positive (48.2%) or even (41.3%) pacing style. Only four athletes (13.8%) matched their executed strategy with their pre-programmed pacing. The ability to match executed and pre-programmed pacing strategy seems not to be related to the athlete’s experience or level of performance. However, high-performance athletes predominantly performed even pacing, while low-performance athletes performed predominantly positive pacing. Interestingly, this difference could not be predicted from the pre-programmed pacing analysis. Together, these findings suggested that a higher performance during race walking might be related to the ability to keep a constant velocity throughout the race, rather than to try to follow the pre-programmed pacing.

In the present study, there was no agreement between pre-programmed and executed pacing. Likewise, a study with cyclists and ultramarathon runners observed many discrepancies between pre-programmed and executed pacing, mainly at the beginning and end sections of the race (24). Cyclists started a 5-km time trial more slowly and finished faster than their prediction. In contrast, during a 100-km head-to-head competition, ultramarathon runners started faster and finished more slowly than their prediction (24). This might indicate that the presence of competitors probably “enforces” a faster start (25). In fact, the presence of an opponent is an important issue during a mass-start event (25), leading to greater velocities at the beginning of a race (26,27). It has been suggested that the pacing template lacks accuracy, indicating greater reliance on momentary pacing decisions rather than pre-planned strategy (24). In the present study, race walkers switched from a pre-programmed negative (slow start) to positive (fast start) pacing, which suggests that the opponents, rather than the pre-programmed strategy, dictated the rhythms at the beginning of the race. Therefore, controlling the velocity at the beginning by prior instruction may be important to preserve a better overall performance and avoid early competitor-induced fatigue.

In the present study, we found no influence of the athletes’ previous experience on their ability to match pre-programmed and executed pacing in real competition. Although this has not been directly tested, a study reporting that final times during consecutive head-to-head marathons within a season is less variable in older than younger runners suggesting that experienced athletes are more consistent with their pre-programmed pacing (18). Our results were contrary to this prediction, perhaps due to the high performance levels of our athletes. All athletes were classified at national and/or international levels. It has been suggested that the multi-faceted pacing skill might be constructed during adolescence in athletes, when experience might be an important factor influencing pacing (28), but has less influence when athletes have reached elite level. Therefore, our findings suggested that high-competitive race walkers adjust their velocity against the challenges imposed during the race, renouncing their pre-programmed pacing strategy, which is independent of their level of experience.

Our results also showed that the performance level of the athletes seems to have no influence on the ability to match pre-programmed with executed pacing. However, an interesting finding was that high-performance athletes adopted mostly an even pacing, while low-performance athletes adopted more aggressive pacing at the beginning (i.e., positive pacing), even though this was not pre-programmed. Previous studies have shown that athletes with a high performance level tend to establish distinct pacing from those of lower level (4,12). Only one study compared level of performance in race walking and found that medalists have the ability to start faster and maintain velocity throughout the race, when compared to non-medalist athletes (12). Our results suggested that during a mass-start event, low-performance athletes changed from even or negative pre-programmed pacing to a more aggressive, positive pacing strategy, perhaps trying to follow the leaders. The effect of this reduces wind resistance and the chances of attracting the judges’ attention for possible disqualification (12,29). This seems to be unsustainable for the entire race and velocity will likely decrease as the race progresses. High-performance athletes, on the other hand, were able to control their start, probably because they employed high velocities at the beginning and sustained them throughout the race.

The strategy to evaluate pre-programmed and executed pacing was inexpensive and easy to apply. Our analyses, however, were based on data from athletes (pre-programmed) and a researcher (executed) using visual identification. It is improbable that this had a strong impact on our results as the athletes had been instructed and familiarized with the five patterns of pacing; the same experience was true for the investigator, who was unaware of the pre-programmed pacing, and classified all executed pacing. Previous studies have demonstrated that pacing during endurance events can be classified only into the five types used in the present study (1,14). In addition, we recruited males and females, and young and older athletes, where both sex and age might have an influence on the capacity to match pre-programmed and executed pacing (18,30). Even with these recognized limitations, this was the first experimental study using highly competitive athletes in real competitions. Thus, our results might be useful to help coaches and athletes to understand the difficulty of matching pre-programmed and executed pacing, and to determine the most efficient pacing strategy for the best performance.

In summary, race walkers did not match their pre-programmed and executed pacing, and this seems to be independent of previous experience and performance level. However, high-performance athletes tended to execute an even pacing regardless if this had or had not been pre-programmed.
